# A theorem proving approach for automatically synthesizing visualizations of flow cytometry data

**DOI:** 10.1186/s12859-017-1662-4

**Published:** 2017-06-07

**Authors:** Sunny Raj, Faraz Hussain, Zubir Husein, Neslisah Torosdagli, Damla Turgut, Narsingh Deo, Sumanta Pattanaik, Chung-Che (Jeff) Chang, Sumit Kumar Jha

**Affiliations:** 1 Computer Science Department, University of Central Florida, Orlando, 32816 Florida USA; 2 School of Computing, University of Utah, Salt Lake City, Utah USA; 3 Department of Pathology, Florida Hospital, Orlando, Florida USA

**Keywords:** Automated synthesis, Symbolic decision procedures, High-fidelity visualization, Biomedical informatics, High-dimensional data, Flow cytometry

## Abstract

**Background:**

Polychromatic flow cytometry is a popular technique that has wide usage in the medical sciences, especially for studying phenotypic properties of cells. The high-dimensionality of data generated by flow cytometry usually makes it difficult to visualize. The naive solution of simply plotting two-dimensional graphs for every combination of observables becomes impractical as the number of dimensions increases. A natural solution is to *project the data from the original high dimensional space to a lower dimensional space while approximately preserving the overall relationship between the data points*. The expert can then easily visualize and analyze this low-dimensional embedding of the original dataset.

**Results:**

This paper describes a new method, SANJAY, for visualizing high-dimensional flow cytometry datasets. This technique uses a decision procedure to *automatically synthesize two-dimensional and three-dimensional projections of the original high-dimensional data while trying to minimize distortion.* We compare SANJAY to the popular multidimensional scaling (MDS) approach for visualization of small data sets drawn from a representative set of benchmarks, and our experiments show that SANJAY produces distortions that are 1.44 to 4.15 times smaller than those caused due to MDS. Our experimental results show that SANJAY also outperforms the Random Projections technique in terms of the distortions in the projections.

**Conclusions:**

We describe a new algorithmic technique that uses a symbolic decision procedure to automatically synthesize low-dimensional projections of flow cytometry data that typically have a high number of dimensions. Our algorithm is the first application, to our knowledge, of using automated theorem proving for automatically generating highly-accurate, low-dimensional visualizations of high-dimensional data.

## Background

Polychromatic flow cytometry is a popular technique for measuring cell properties. These properties include DNA and RNA content, intracellular phosphoproteins, cytokines, and cell-surface proteins [1]. In this technique, multiple fluorescent dyes corresponding to desired phenotypic observables are first used to label cell components. The cells are then made to flow through a detector in a single file, and their fluorescence is measured. Flow cytometry has applications in lymphoma phenotyping, cell sorting, HIV, stem cell identification, tumor ploidy, and solid organ transplantation [2]. Unlike traditional techniques that take the statistical average of a sample, flow cytometry works on a per-cell basis. Therefore, it can be used to analyze multiple phenotypic observables simultaneously and at a rate of thousands of cells per second [2].

Data generated from flow cytometry analysis enables an experimental scientist to identify rare properties of small groups of cells that would not have been traditionally possible through observing the average properties of all cells in a sample. The analysis of such groups of rare cells becomes even more important if we consider the case of cancer patients, where early detection of rare cell phenotypes might be key to saving a patient. Similarly, the absence of rare phenotypic observables in a sample may suggest the termination of certain medication or treatments in subjects already suffering from cancer. The analytical power of flow cytometry brings with it two major barriers that need to be overcome for its effective and widespread employment in scientific practice:

(i) Since polychromatic flow cytometry can observe multiple phenotypes simultaneously, this leads to data with multiple dimensions. According to various cognitive processing studies, the data analysis capacity of human beings is limited, on average, to about four dimensions that can be processed in parallel [3, 4]. Therefore, flow cytometry techniques that often produce data in 10 or more dimensions cannot be easily analyzed by human experts.
(ii) Polychromatic flow cytometry is used to generate data about individual cells; so, the size of the data obtained from the analysis is usually very large. The dataset can consist of millions of data points per sample which is well beyond the cognitive memory limit of human beings [5]. Standard statistical methods that involve summarization negate the advantages of flow cytometry by making the result similar to traditional measurement methods that produce observables only on the average property of a sample. Statistical methods may lead to loss of small but significant details needed to detect rare but interesting cellular phenotypes.

We address these problems by designing a new automated technique for synthesizing low-dimensional visualizations of flow cytometry data. This paper makes the following contributions:

(i) We describe SANJAY – a new algorithmic approach for automatically synthesizing 2D and 3D visualizations of high-dimensional flow cytometry data. SANJAY’s main contribution is to employ automated algorithmic synthesis techniques [6, 7] and symbolic decision procedures [8] to create low-dimensional projections of high-dimensional data that can be easily visualized.
(ii) This algorithmic projection approach approximately preserves the original relationship between the points in the high-dimensional space. This algorithm avoids stastical summarization thus minimizing the loss of small but rare events.
(iii) We compare SANJAY to the popular multi-dimensional scaling (MDS) algorithm on small high-dimensional data sets and show that our projections produce distortions that are on average 2.56 times smaller than those produced by MDS (see Table 1).

**Table 1:** Distortions produced by the MDS approach and SANJAY when 10 randomly chosen high-dimensional data points from 30 flow cytometry datasets were projected onto two dimensions

Dataset	Maximum	Maximum	Ratio of	Dataset	Maximum	Maximum	Ratio of
ID	distortion	distortion	maximum distortions	ID	distortion	distortion	maximum distortions
	for MDS	for SANJAY	MDS/SANJAY		for MDS	for SANJAY	MDS/SANJAY
1	3197.8	1000	3.197	16	3150.4	1200	2.625
2	2711.1	1200	2.259	17	2497.2	1100	2.270
3	1953.0	1000	1.953	18	2925.5	1400	2.089
4	2917.2	1200	2.431	19	3813.3	1300	2.933
5	3483.5	1400	2.488	20	3700.8	1300	2.846
6	2925.9	1100	2.659	21	3011.8	1200	2.509
7	4233.0	1800	2.351	22	3252.4	1000	3.252
8	2898.0	1300	2.229	23	3381.4	1200	2.817
9	1876.7	1300	1.443	24	2963.9	1100	2.694
10	4314.1	1500	2.876	25	3428.3	1600	2.142
11	3543.6	1400	2.531	26	2712.2	1200	2.260
12	2449.8	1300	1.884	27	3679.7	1500	2.453
13	3835.2	1500	2.556	28	3286.0	1200	2.738
14	4153.3	1000	4.153	29	2449.7	1000	2.449
15	2858.6	1000	2.858	30	4160.0	1400	2.971

The maximum distortion produced by SANJAY was, on average, 2.56 times less than that produced by MDS

### Automated gating of flow cytometry data

Machine learning methods have been deployed for automatically labeling subpopulations of cells in flow cytometry data sets – a process popularly referred to as gating. In particular, supervised and semi-supervised machine learning algorithms [9, 10] have been extensively investigated for automatically identifying related cells.

*Sequential gating* [11] enables two-dimensional visualization of any two colors or dimensions of data from a polychromatic flow cytometer. The human expert then attempts to manually identify subsets of cells that correspond to the same subpopulation. While the process is computationally simple, the result is highly subjective and depends on the intuition of the oncologist. Further, an *n*-dimensional flow cytometry data has *n*×(*n*−1)/2 possible two-dimensional visualizations. Thus, a 20-color polychromatic flow cytometer will produce 190 different 2-dimensional visualizations and it is a cognitive challenge for a human expert to verify clinical or experimental conjectures against all 190 visualizations obtained from a biological sample.

Probability binning [12] is an unsupervised quantitative methodology for analyzing polychromatic flow cytometry data that identifies the difference between the distribution of cells in a given sample and a standard control sample. Frequency difference gating [13] extends this approach by enabling multidimensional gating of the bins identified by the probability-binning algorithm that contain the largest differences between the given and the control sample.

Cluster analysis methods [14, 15] employ varying levels of expression of antigens to construct subsets of cells that share the same combination of fluorochromes markers. While the technique is unsupervised, the result is only a semi-quantitative two-dimensional visual description (such as a heat map) of the data set and still needs to be interpreted subjectively by an expert for biological correctness. Standard machine learning algorithms such as k-means [16] and expectation maximization [17] have been applied to perform cluster analyses of polychromatic flow cytometry data.

The most popular clustering algorithm that operates by building and refining partitions is the k-means algorithm [18, 19]. The popular k-means algorithms have also been applied to flow cytometry data [17]. The k-means algorithm requires three inputs from the user: the number of clusters, an initial cluster assignment, and a metric to measure distance between data points. As the k-means algorithms converge only to one of the local minima, different initializations of the k-means algorithm can lead to different final clustering of the data. Such sensitivity to initial conditions is undesirable for an objective flow cytometry data exploration framework.

Principal Component Analysis (PCA) is a particularly popular approach for generating two-dimensional visualizations of flow cytometry data [15]. However, low-dimensional visualizations lose a lot of information because of the low correlation between different fluorochromes, and such plots mostly serve as an exploratory tool in the hands of well-trained experts.

In our recent work [20], we have proposed the use of complex network models and their topological properties for discriminating between cancer and normal patients. In our approach, each node in the complex network corresponds to the measurements obtained from a single cell and an edge between two nodes exists if the Euclidean distance between them is smaller than a threshold. The evolution of the network through time can be derived by studying periodically acquired patient samples. By constructing such complex network models for multiple normal patients, we propose to develop a stochastic generative model that describes the flow cytometry data for normal patients. In particular, topological properties such as number of connected components, edge density, number of clusters, etc. are studied. The goal of our stochastic generative modeling is to capture the natural diversity that occurs in the normal patient population (age, race, gender, BMI), and thereby compute the probability that a given flow cytometry sample does not arise from this stochastic generative model. Rare behavior identification algorithms, including our own work [21], can then be employed to compute the probability that a given flow cytometry sample indicates the presence of a physiological anomaly in a patient.

### Decision procedures

To the best of our knowledge, our current work is the first effort towards the application of symbolic decision procedures for the algorithmic synthesis of projections from high-dimensional data to low-dimensional visualizations. In 1929, Mojzesz Presburger introduced a first-order theory of arithmetic for natural numbers with addition and equality – a consistent, complete and decidable fragment of logic [22]. Fifty years later, Robert Shostak presented an algorithm for deciding quantifier-free Presburger arithmetic that permits arbitrary uninterpreted functions [23]. More recently, a number of decision procedures for verifying various decidable fragments of logic involving arithmetic and function symbols have been proposed and implemented using the popular SMTLIB standard [24]. In particular, a number of decision procedures for bit-vectors involving arithmetic and logical operations have been successfully implemented [25, 26]. Many of these approaches build upon the foundation work of Martin Davis, Hilary Putnam, George Logemann and Donald W. Loveland who introduced the DPLL algorithm for checking the satisfiability of propositional logic formulas in 1962 [27]. We show that our approach based on bit-vector decision procedures outperforms classical multi-dimensional scaling approach – at least on small high-dimensional data sets – by consistently creating projections with at least 80% less distortion.

### Some notations and definitions

We now recall some basic ideas relevant to our use of decision procedures for the automated synthesis of visualizations.

#### **Definition 1**

*(Basic bit-vector operations)* A bit-vector is a vector of Boolean values of a given length. Given two bit-vectors, their bitwise logical operations are performed by applying the logical operation to the corresponding bits of the bit-vectors.

$$\begin{array}{@{}rcl@{}} &&\lnot x = \lambda i \in \{0, 1, \ldots, l-1\}.\lnot x_{i}\\ &&x \lor y = \lambda i \in \{0, 1, \ldots, l-1\}. \left(x_{i} \lor y_{i}\right)\\ &&x \land y = \lambda i \in \{0, 1, \ldots, l-1\}. \left(x_{i} \land y_{i}\right)\\ \end{array} $$

The above equations define the formal semantics of bit-vector NOT, OR, and AND operations. Similarly, arithmetic operations such as addition and subtraction can be defined on bit-vectors by extending the standard definition of these operations from the decimal to the binary representation.

#### **Definition 2**

*(Bit-vector concatenation)* Two bit-vectors of length *l* and *l* ^′^ can be concatenated into a single bit-vector of length *l*+*l* ^′^.

$$ \begin{aligned} xy &= \lambda i \in \left\{0, 1, \ldots, l+l^{\prime}-1\right\}. b_{i}\ \text{where},\\ b_{i} &= \left\{\begin{array}{ll} x_{i} & \text{if \(i < l\)}\\ y_{i-l} & \text{otherwise}. \end{array}\right. \end{aligned} $$

Relational operations on bit-vector are defined similarly, using both signed and unsigned interpretations [24]. As these formulas naturally arise in software and hardware verification, several solvers for bit-vector decision procedures are widely deployed. The top solvers in the 2015 SMT-COMP competition for bit-vectors include Boolector, CVC4, STP, Yices, Mathsat and Z3. Most of these solvers use a combination of bit-blasting and rewriting to translate the bitvector decision problem into a combination of lemmas that can be discharged using results from number theory and satisfiability solving [28].

#### **Definition 3**

*(Distortion)* Distortion is defined as the change of distance between two points when they are projected from a high-dimensional space to a lower dimension. Let the distance between points *x* and *y* in the original space be *d*(*x*,*y*). Let the projections of *x* and *y* in the lower dimension space be *x* ^′^ and *y* ^′^ respectively. Let *d*(*x* ^′^,*y* ^′^) be the distance between the projected points. The distortion due to this projection is defined by:

$$ distortion(x,y) = \left|d\left(x',y'\right)-d\left(x,y\right)\right| $$

## Methods

### Graph representation of flow cytometry data

**Fig. 1:**
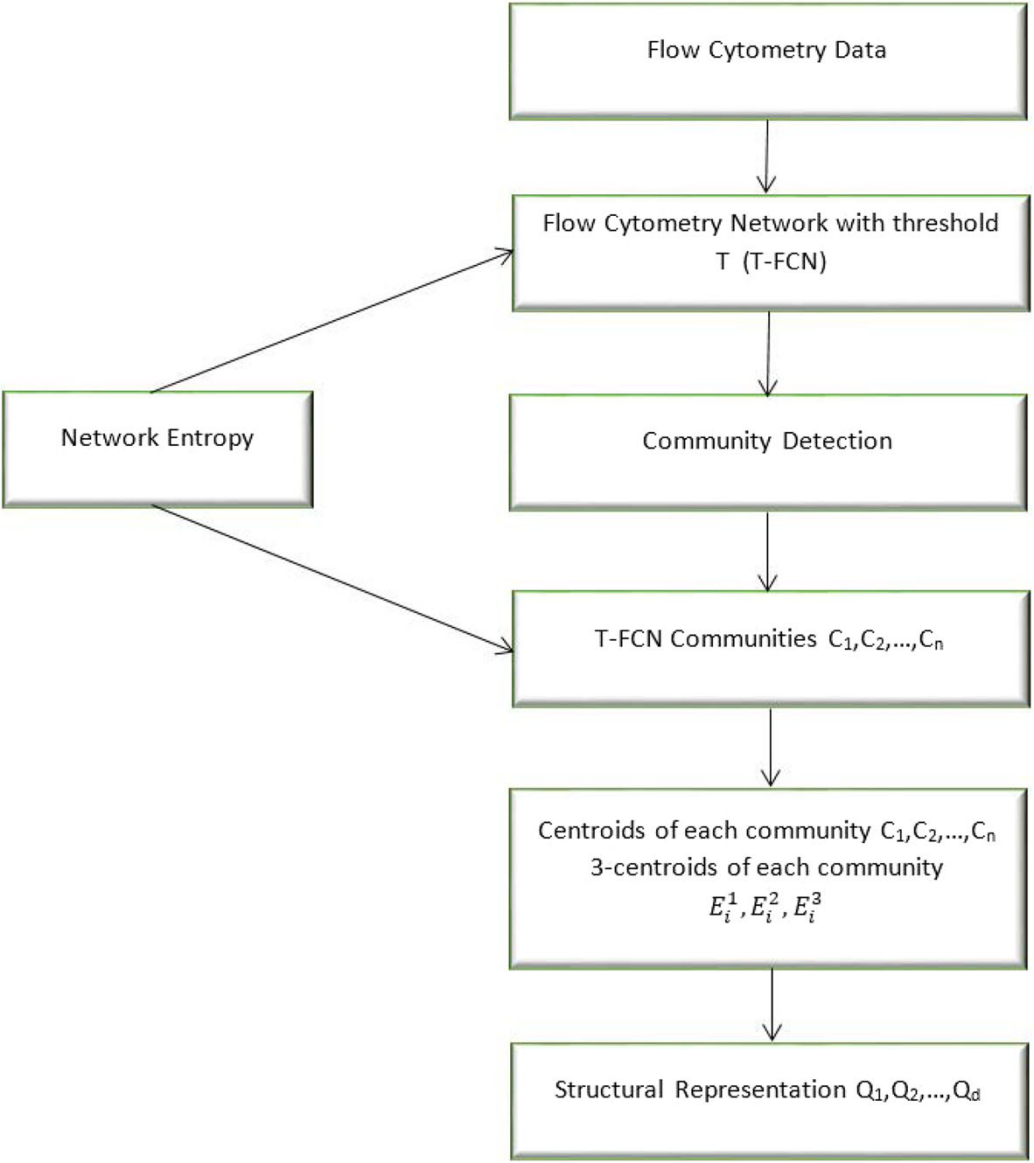
Steps for generating the structural representation of flow cytometry data for use in the SANJAY visualization synthesis technique

There is an inherent complex network structure in polychromatic flow cytometry data arising from the well-governed biological process of cell differentiation. Using our earlier approach [20], we can build a complex network representation of the observed flow cytometry data set. We follow the steps outlined in Fig. 1 to create a structural representation of flow cytometry data.

#### **Definition 4**

*(Flow Cytometry Network)* Given *N* *m*-dimensional data points representing *N* cells, each representing *m* observed properties measured by a polychromatic flow cytometer, the flow cytometry network with threshold *T* (a T-FCN) is a graph *G*=(*V*,*E*) where *V* is the set of nodes and *E* is the set of edges, such that:

- a node *v*∈*V* denotes the *m* quantities measured for a single cell, i.e. *v*=(*v*_0_,*v*_1_,…,*v*_*m*−1_), and
- (*v*,*v* ^′^)∈*E* if and only if $||\left (v_{0}, \ldots, v_{m-1}\right) - \left (v^{\prime }_{0}, \ldots, v^{\prime }_{m-1}\right)|| \leq T$.

The second property above specifies that there’s an edge between two nodes (i.e. between data points representing a pair of cells), when the Manhattan distance between them is less than threshold *T*. Recall that the Manhattan distance between vectors *v*=(*v*_0_,…,*v*_*m*−1_) and *u*=(*u*_0_,…,*u*_*m*−1_) is defined to be $\sum ^{m-1}_{i=0} \left \lvert v_{i} - u_{i} \right \rvert $.

Given flow cytometry data, a T-FCN (flow cytometry network) is determined by the threshold *T* that is used to decide whether two nodes in the flow cytometry network are connected by an edge in the T-FCN. The threshold *T* is typically learned from experimental data. As *T* is varied from *∞* to 0, the T-FCN goes from being a clique of *N* nodes to being a network with *N* components – each node being a component by itself. The variation in *T* causes changes in the distribution of the topological properties.

Using information theoretic arguments [29, 30], we can compute the value of *T* that maximizes the information content or entropy of the distribution of the topological properties. Thus, the generated T-FCN is the most informative network describing the flow cytometry data set.

### Community detection in flow cytometry data

Several existing algorithms are capable of identifying communities in large complex networks [31]. Due to the massive size of the network generated by a typical flow cytometry dataset, one can readily rule out the use of matrix and spectral graph theory based methods. Modularity based methods are known to be biased against small communities and are hence not a method of choice for identifying communities in flow cytometry networks, where small communities may represent rare but interesting anomalies [32].

Keeping in mind our high-assurance requirement for biomedical applications, and the large size of flow cytometry datasets, we suggest the use of a parallel version of the Walktrap algorithm for community detection [20] in our flow cytometry networks [33]. The main idea behind Walktrap approach is based on the intuition that random walks of a graph must be trapped in densely connected communities of the T-FCN that are only sparsely connected to the rest of the network. As several random walks can be instantiated in parallel on multiple processing nodes, the approach is readily deployable on large supercomputing clusters [34].

### Structural representation of flow cytometry networks

Each flow cytometry data set is represented by a T-FCN that maximizes the information content of the network. A flow cytometry network T-FCN is then decomposed into a number of communities *C*_1_,…,*C*_*n*_, using methods described in the previous section where each *C*_*i*_ is itself a T-FCN. The centroid of a community can serve as a surrogate representing the approximate position of all the points in the community. To preserve the relative position of the communities, we compute the centroids *O*_1_,…,*O*_*n*_ of the communities and seek to approximately preserve the distance between these centroids. In order to preserve the geometry of the individual communities, we also must compute the 3-centroids $E^{1}_{i}, E^{2}_{i}, E^{3}_{i}$ for each community *C*_*i*_ when projecting into two dimensions (and 4-centroids when projecting into three dimensions). To calculate 3-centroids of a community *C*_*i*_, we break the community into 3 component communities $C^{1}_{i}, C^{2}_{i},C^{3}_{i}$ using k-means clustering algorithm where the input k for the k-means algorithm is equal to 3. We then calculate one centroid for each of the 3 component communities for a total of 3 component centroids $E^{1}_{i}, E^{2}_{i},E^{3}_{i}$ corresponding to each community *C*_*i*_. For projecting onto two dimensions, the set of points $\left \{O_{1}, E^{1}_{1}, E^{2}_{1}, E^{3}_{1}, O_{2}, E^{1}_{2}, E^{2}_{2}, E^{3}_{2}, \ldots, O_{n}, E^{1}_{n}, E^{2}_{n}, E^{3}_{n}\right \}$, that we will also denote by *Q*_1_,…,*Q*_*d*_ where *d*=4*n* and *n* is the number of communities in the T-FCN, serves as a structural representation of the flow cytometry network.

### Automated synthesis of projections using decision procedures

Given the structure-defining points {*Q*_1_,…,*Q*_*d*_} = $\left \{O_{1}, E^{1}_{1}, E^{2}_{1}, E^{3}_{1}, O_{2}, E^{1}_{2}, E^{2}_{2}, E^{3}_{2}, \ldots, O_{n}, E^{1}_{n}, E^{2}_{n}, E^{3}_{n}\right \}$ in *m* dimensions, SANJAY synthesizes an embedding {*R*_1_,…,*R*_*d*_} of the points in two-dimensional or any other lower dimensional space that approximately preserves the pairwise Manhattan distances between these points up to an error of *ε*>0. The following expression specifies relationship between the original points *Q*_1_,…,*Q*_*d*_ and the synthesized lower-dimensional projection *R*_1_,…,*R*_*d*_ with respect to the distortion *ε*:

$$\begin{array}{@{}rcl@{}} \exists R_{1}, R_{2} \ldots, R_{d}, \forall i, j \in \{1, \ldots d\},\\ \bigwedge_{i,j, i \neq j} \lvert\lvert R_{i} - R_{j} \rvert\rvert \leq \lvert\lvert Q_{i} - Q_{j}\rvert\rvert + \epsilon\\ \bigwedge_{i,j, i \neq j} \lvert\lvert R_{i} - R_{j} \rvert\rvert \geq \lvert\lvert Q_{i} - Q_{j}\rvert\rvert - \epsilon\\ \end{array} $$

To help in discussing our projection algorithm, we now state, without proof, a lemma that describes the requirement for the location of a point in 2D or 3D space to be fixed.

#### **Lemma 1**

(Fixing points in two and three dimensions) For any given point in two-dimensional space, its distance from three unique points uniquely identify its coordinates. Similarly, for any point in three-dimensional space, its distance from four unique points uniquely identify its coordinates *[* 35 *]*.

Therefore, the two-dimensional projection of all points in a community *C*_*i*_ can be obtained using the 2D projections of the 3-centroids $E^{1}_{i}, E^{2}_{i}, E^{3}_{i}$ of that community. Similarly, the three-dimensional projections of the points in a community can be obtained from the projections of the 4-centroids $E^{1}_{i}, E^{2}_{i}, E^{3}_{i}, E^{4}_{i}$ of the community.

However, a direct translation of the problem to bit-vector decision procedures involves a tradeoff between computational tractability and the accuracy of the obtained projections. Large values of *ε* lead to decision problems that can be readily solved by decision procedures but correspond to poor projections. Small *ε* values represent high-quality distance-preserving projections but create computationally challenging instances of the decision problem.

The SANJAY algorithm solves the problem by using an *iterative refinement* to derive the points *R*_1_,*R*_2_,…,*R*_*d*_ in the lower-dimensional space from the pairwise distances between the points *Q*_1_,…,*Q*_*d*_ in the higher dimension. The algorithm starts by synthesizing the highest-order bit in the bit-vector representation of these points, and then searches for the other bits.



SANJAY is formally illustrated in Algorithm 1. The algorithm accepts the pairwise distances *D*_*i*,*j*_(1≤*i*,*j*,≤*d*) between every pair of *d* points as an input. It also accepts two other inputs: the length *b* of the bit-vector representing the projected points to be synthesized and the number of bits *l* that should be learned in every iteration of the projection synthesis loop.

In Algorithm 1, a point *Q*_*i*_ is represented by the bit vector representation $\left (P^{s}_{x_{i}}a^{r},P^{s}_{y_{i}}b^{r}\right)$ where $P^{s}_{x_{i}}a^{r}$ is the *x*-coordinate and $P^{s}_{y_{i}}b^{r}$ is the *y*-coordinate. The $P^{s}_{x_{i}}$ and $P^{s}_{y_{i}}$ are the parts of the vector that have been calculated by the algorithm, the *a* ^*r*^ and *b* ^*r*^ are the parts of the vector that have still not been calculated. When all the bits of any vector *a* ^*r*^ are 1 then we denote it by 1^*r*^ similarly when all the bits of the vector are 0 we denote it by 0^*r*^. The bit vector *a* ^*r*^ has the property that 0^*r*^≤*a* ^*r*^≤1^*r*^. So, any point *Q*_*i*_ with representation $\left (P^{s}_{x_{i}}a^{r},P^{s}_{y_{i}}b^{r}\right)$ can take all the values within the square with corners $\left (P^{s}_{x_{i}}0^{r},P^{s}_{y_{i}}0^{r}\right),\!\left (P^{s}_{x_{i}}0^{r},P^{s}_{y_{i}}1^{r}\right), \left (P^{s}_{x_{i}}1^{r},P^{s}_{y_{i}}0^{r}\right),\left (P^{s}_{x_{i}}1^{r},P^{s}_{y_{i}} 1^{r}\right)$.

Algorithm 1 initializes the length *s* of the projected points to 0. The algorithm also initializes the length *r* of the remaining bit-vectors to be synthesized with the value *b*. This means that the point *P*_*i*_ can take all the values within the square denoted by the points (1^*b*^,1^*b*^),(1^*b*^,0^*b*^),(0^*b*^,1^*b*^),(0^*b*^,0^*b*^). This square spans the whole search space, which implies that at the start of the first iteration, the point *P*_*i*_ can be found anywhere in this search space.

A bit-vector decision procedure then searches for a better approximation of the projected point by searching for the next *l* higher order bits $A^{1}_{1}, A^{1}_{2},\ldots, A^{1}_{l}$ in the binary representation of the projection of the points by solving the following decision problem:

1 $$ B_{i} = \left\lVert \left(P^{s}_{x_{i}}A^{l}_{x_{i}}a^{r}, P^{s}_{y_{i}}A^{l}_{y_{i}}b^{r}\right) - \left(P^{s}_{x_{j}}A^{l}_{x_{j}}c^{r}, P^{s}_{y_{j}}A^{l}_{y_{j}}d^{r}\right) \right\rVert^{2}  $$

2 $$ (1-\epsilon) D_{i,j}^{2} \leq \quad \max_{a,b,c,d \in \{0,1\}}{} B_{i} \quad \leq (1+\epsilon) D_{i,j}^{2}  $$

Each iteration of the algorithm breaks down the previous square into 2^2*l*^ sub-squares in which the point *P*_*i*_ can be found and Eq. 2 using bit vector decision procedure selects the best possible sub-square for the point *P*_*i*_. At the end of the iteration, each of the points is projected to a sub-square with the diagonal $\left (P^{s}_{x_{i}}A^{l}_{x_{i}}0^{{r} - {l}},P^{s}_{y_{i}}A^{l}_{y_{i}}\, 0^{{r} - {l}}\right)$ and $\left (P^{s}_{x_{i}}A^{l}_{x_{i}}1^{{r} - {l}},P^{s}_{y_{i}}\,A^{l}_{y_{i}}1^{{r} - {l}}\right)$, where $P^{s}_{x_{i}}$ and $P^{s}_{y_{i}}$ denote bit vectors of *s* bits, $A^{l}_{x_{i}}$ and $A^{l}_{y_{i}}$ denote bit vectors of *l* bits, and 0^*r*−*l*^ is a zero bit vector of *r*−*l* bits.

As the algorithm iterates, it builds finer abstractions of the bit-vector representation of the points being projected. When the algorithm has computed *b* number of bits in the bit-vector representation of the projected points, it assigns the generated bit-vectors to the output *R*_1_,…,*R*_*d*_.

## Results and discussion

We performed our experimental evaluation on a 64-core 1.40GHz AMD Opteron(tm) 6376 processor with 64 GB of RAM. We analyzed 30 flow cytometry data sets – each of them having 12 dimensions.

For each dataset, we used MDS [36], random projections [37] and our SANJAY technique, to search for two-dimensional projections of 10 randomly selected data points from the original high-dimensional data, while seeking to maintain the original inter-point distances. We then computed the maximum and the average distortion of the projections produced by all three techniques.

**Fig. 2:**
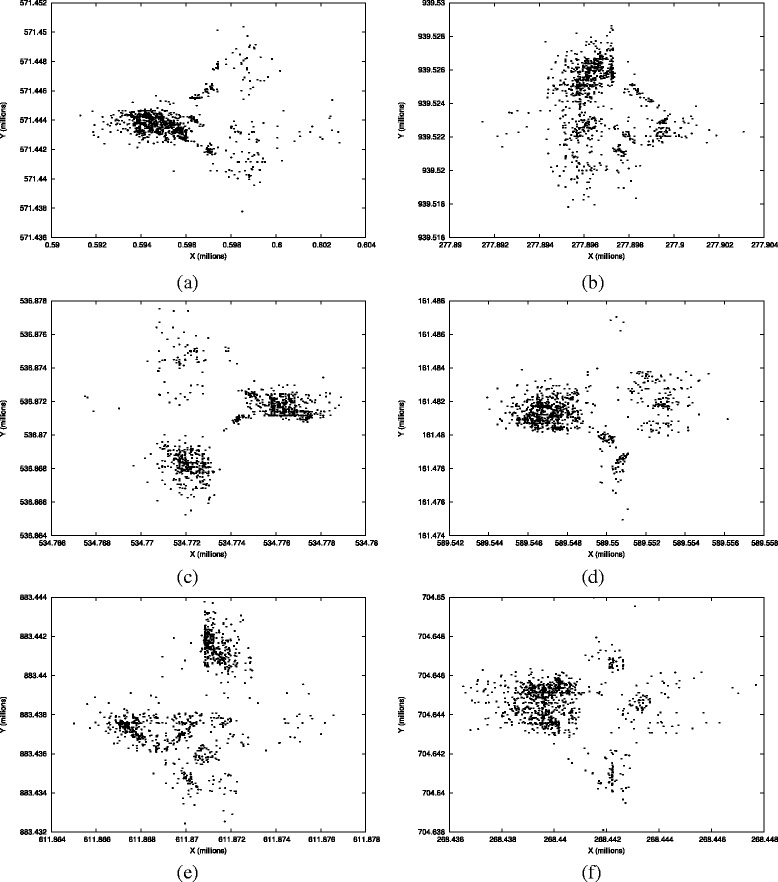
Plots of the two dimensional projections synthesized by the SANJAY algorithm for 1000 randomly chosen data points from 6 flow cytometry datasets (dataset IDs 9, 24, 11, 14, 17, and 5 respectively in Table 1). For these and 24 other flow cytometry datasets, Table 1 lists the maximum distance distortion when 12-dimensional flow cytometry data is projected onto two dimensions, and Table 2 lists the average distortions

The comparison between SANJAY and MDS is presented in Tables 1 and 2. SANJAY performed at least 1.44 times better and sometimes as much as 4.15 times better than MDS in terms of minimizing the maximum distance distortion among all the projected points. The average distortions due to SANJAY were as much as 2.33 times lower than those produced using the MDS approach. Figure 2 shows the results of using SANJAY to project 1000 randomly chosen points from 6 of the 30 flow cytometry datasets discussed above.

**Table 2:** Average distortions produced by the MDS approach and SANJAY when 10 randomly chosen high-dimensional data points from 30 flow cytometry datasets were projected onto two dimensions

Dataset	Average distortion	Average distortion	Dataset	Average distortion	Average distortion
ID	for MDS	for SANJAY	ID	for MDS	for SANJAY
1	1042.4	540.8	16	1034.4	733.8
2	1024.4	653.3	17	919.5	623.0
3	649.2	537.5	18	1056.8	822.4
4	897.4	765.3	19	1117.4	757.5
5	1089.6	806.3	20	989.5	773.6
6	1069.4	634.0	21	1057.5	684.8
7	1374.4	1010.7	22	1412.6	605.7
8	949.8	709.4	23	915.0	712.8
9	765.9	752.5	24	824.3	741.1
10	1011.7	892.9	25	1178.1	1033.5
11	1050.4	882.8	26	949.2	713.3
12	1050.3	760.0	27	1114.2	833.6
13	1241.7	849.7	28	935.4	611.7
14	985.7	613.4	29	1004.8	561.3
15	1249.6	612.4	30	1178.4	874.1

The comparison between SANJAY and random projections is shown in Tables 3, and 4. When compared with random projections, SANJAY performed 7.02 times better at minimizing the maximum pairwise distortion among points. We envision that such automatically generated visualizations can be used to identify patients whose flow cytometry data indicates a significant number of cells showing abnormal behavior.

**Table 3:** Maximum distortions produced by SANJAY and Random Projections technique when 10 randomly chosen high-dimensional data points from 30 flow cytometry datasets were projected onto two dimensions

Dataset	Maximum	Maximum distortion	Ratio of maximum	Dataset	Maximum	Maximum distortion	Ratio of maximum
ID	distortion	for random	distortions	ID	distortion	for random	distortions
	for SANJAY	projections	RP/SANJAY		for SANJAY	projections	RP/SANJAY
1	1000	4069	4.07	16	1200	6732	5.61
2	1200	4179	3.48	17	1100	4298	3.90
3	1000	3982	3.98	18	1400	4922	3.51
4	1200	5289	4.40	19	1300	6719	5.16
5	1400	5045	3.60	20	1300	5583	4.29
6	1100	5092	4.62	21	1200	5311	4.42
7	1800	5364	2.98	22	1000	4447	4.44
8	1300	3566	2.74	23	1200	4731	3.94
9	1300	4357	3.35	24	1100	6251	5.68
10	1500	4262	2.84	25	1600	5919	3.69
11	1400	4945	3.53	26	1200	5385	4.48
12	1300	4370	3.36	27	1500	4886	3.25
13	1500	4747	3.16	28	1200	5884	4.90
14	1000	7029	7.02	29	1000	5398	5.30
15	1000	6161	6.16	30	1400	3900	2.78

**Table 4:** Average distortions produced by SANJAY and Random Projections when 10 randomly chosen high-dimensional data points from 30 flow cytometry datasets were projected onto two dimensions

Dataset	Average	Average distortion	Ratio of average	Dataset	Average	Average distortion	Ratio of average
ID	distortion	for random	distortions	ID	distortion	for random	distortions
	for SANJAY	projections	RP/SANJAY		for SANJAY	projections	RP/SANJAY
1	540.8	1289.2	2.38	16	733.8	1791.5	2.44
2	653.3	1226.5	1.87	17	623.0	1361.3	2.18
3	537.5	1095.5	2.03	18	822.4	1480.3	1.80
4	765.3	1637.1	2.13	19	757.5	1912.7	2.52
5	806.3	1654.7	2.05	20	773.6	1806.0	2.33
6	634.0	1555.5	2.45	21	684.8	1535.2	2.24
7	1010.7	1608.8	1.59	22	605.7	1440.1	2.37
8	709.4	1111.8	1.56	23	712.8	1355.4	1.90
9	752.5	1439.5	1.91	24	741.1	1944.2	2.62
10	892.9	1376.7	1.54	25	1033.5	1943.4	1.88
11	882.8	1578.5	1.78	26	713.3	1762.9	2.47
12	760.0	1395.6	1.83	27	833.6	1519.0	1.82
13	849.7	1363.1	1.60	28	611.7	1648.0	2.69
14	613.4	2084.7	3.39	29	561.3	1513.4	2.70
15	612.4	1916.6	3.12	30	874.1	1047.5	1.19

## Conclusion

In this paper, we described a new algorithmic technique for automatically generating low dimensional visualizations of high-dimensional flow cytometry data. We used symbolic decision procedures to exhaustively search for low-dimensional projections in a finite, discretized search space. Our results show that visualizations synthesized using our technique (SANJAY) were better than those produced by the multi-dimensional scaling and random projections approaches in terms of the maximum distortion in the pairwise distances. The results themselves are not surprising as symbolic decision procedures are often used for solving optimization and search problems.

Our experimental results have so far focussed on small fragments of high-dimensional flow cytometry data sets. However, their use in generating such high-fidelity visualizations has not been reported before. In the future, we plan to investigate how our approach can be extended to visualize large data sets while establishing provable bounds on the approximation errors.
